# Deep Learning-Based Objective Quantification of Nasopharyngeal Endoscopic Findings for Standardized Assessment of Inflammation

**DOI:** 10.3390/diagnostics16132015

**Published:** 2026-06-27

**Authors:** Manabu Mogitate, Hirobumi Ito, Yoshihiro Ohno, Noriko Nishiwaki, Yusei Yamaguchi, Momoki Fujikawa, Akira Fukuo, Yuko Sasaki, Yoshiyuki Watanabe, Kota Wada

**Affiliations:** 1 Otolaryngology, Mogitate ENT Clinic, Kawasaki 213-0011, Japan; sasako_0114@yahoo.co.jp; 2 Otolaryngology, Ito ENT Clinic, Funabashi 274-0824, Japan; hirobu@poppy.ocn.ne.jp; 3 Otolaryngology, Ohno ENT Clinic, Fussa 197-0024, Japan; ohnoentclinic@t-net.ne.jp; 4 Otolaryngology, Motohashi ENT Clinic, Mishima 411-0831, Japan; ent.motohasi@space.ocn.ne.jp; 5 Otolaryngology–Head and Neck Surgery, Toho University Omori Medical Center, Tokyo 143-8541, Japan; yusei.yamaguchi@med.toho-u.ac.jp (Y.Y.); momoki.fujikawa@med.toho-u.ac.jp (M.F.); akira.fukuo@med.toho-u.ac.jp (A.F.); ponponta@marianna-u.ac.jp (Y.W.); wadakota@med.toho-u.ac.jp (K.W.); 6 Department of Internal Medicine, Kawasaki Rinko General Hospital, Kawasaki 210-0806, Japan

**Keywords:** nasopharyngitis, endoscopy, artificial intelligence, deep learning, inflammation, epipharyngeal abrasive therapy

## Abstract

**Background/Objectives:** Nasopharyngeal inflammation is commonly evaluated through visual inspection of endoscopic findings, which remains subjective and prone to interobserver variability. This study aimed to develop and validate a deep learning-based system for objective quantification of key nasopharyngeal endoscopic findings. **Methods:** A total of 200 endoscopic videos were retrospectively analyzed as an independent evaluation dataset, while a separate annotated dataset of 279 cases was used for model training. Four findings—mucosal color tone, swelling, mucus or crust adhesion, and bleeding after abrasion—were scored by expert otolaryngologists using a three-point scale, and their sum was used as a composite reference severity score (Y8, range 0–8). A convolutional neural network generated continuous probability outputs for each finding, which were aggregated into a composite score (S8). **Results:** For the primary threshold (Y8 ≥ 3), the AI-derived score demonstrated strong agreement with expert consensus (AUC 0.874). A predefined rule-based diagnostic criterion also showed comparable discriminative performance (AUC 0.851). **Conclusions:** Deep learning-based quantification provides an objective and reproducible method for evaluating nasopharyngeal endoscopic findings. This approach may enable standardized assessment of inflammation and support more consistent clinical decision-making, particularly for identifying clinically relevant inflammation, while its ability to stratify higher severity levels is more limited.

## 1. Introduction

The nasopharynx plays a critical role in upper airway immunity, supported by the presence of nasopharyngeal lymphoid tissue within Waldeyer’s ring [1]. Persistent inflammation in this region is clinically associated with a variety of symptoms, including throat discomfort, postnasal drip, chronic cough, and systemic complaints, which are commonly encountered in daily otolaryngology practice [2]. Direct visualization of the nasopharynx using endoscopy is currently the only practical method for evaluating mucosal conditions in detail, as other imaging modalities do not provide sufficient resolution for assessing subtle inflammatory changes [3].

Despite its clinical importance, the evaluation of nasopharyngeal inflammation is largely subjective and lacks standardized diagnostic criteria. Endoscopic findings such as mucosal color tone, swelling, mucus or crust adhesion, and bleeding after abrasion are widely recognized as key indicators of inflammation. However, their interpretation relies heavily on visual judgment, and considerable interobserver variability exists even among experienced clinicians [4,5,6]. Furthermore, these findings are inherently continuous rather than discrete, yet they are typically categorized using ordinal scales in clinical practice, which may oversimplify subtle but clinically relevant differences in inflammatory severity.

This lack of standardization has important clinical implications. In particular, decisions regarding therapeutic interventions are often guided by subjective endoscopic assessments. For example, the indication for epipharyngeal abrasive therapy (EAT), a treatment widely used in clinical practice, is frequently based on the presence and severity of specific endoscopic findings. Variability in interpretation may therefore directly influence treatment decisions and outcomes. Establishing an objective and reproducible method for evaluating nasopharyngeal inflammation is essential for improving both clinical decision-making and comparability across studies.

This limitation has been reported across multiple clinical fields [4,5,6]. In recent years, deep learning techniques have demonstrated promising performance in medical image analysis, particularly in endoscopic imaging, where they have been successfully applied to lesion detection, classification, and severity grading [7,8,9,10]. These approaches enable automated extraction of complex imaging features and provide objective, quantitative outputs that may reduce variability and improve diagnostic consistency. Recent advances in artificial intelligence have extended the application of deep learning to endoscopic evaluation across various anatomical regions, including the gastrointestinal tract, larynx, and lower airway [11,12]. These studies have shown that deep learning models can achieve performance comparable to or exceeding that of human experts in specific tasks. However, application of such methods to the nasopharynx remains limited. To date, no studies have systematically quantified multiple nasopharyngeal endoscopic findings and integrated them into a unified framework for assessing overall inflammatory activity. In this study, we aimed to develop and validate a deep learning-based system for the objective quantification of key nasopharyngeal endoscopic findings. The proposed framework generates continuous probability outputs for individual inflammatory features and aggregates them into a composite severity score. We further evaluated the agreement between the AI-derived score and expert consensus, as well as its performance in reproducing a clinically motivated rule-based diagnostic criterion. By providing a quantitative and reproducible method for endoscopic evaluation, this approach has the potential to standardize the assessment of nasopharyngeal inflammation and support clinical decision-making.

## 2. Materials and Methods

### 2.1. Study Design and Ethics

This retrospective observational study was conducted to develop and validate a deep learning-based system for the objective assessment of nasopharyngeal endoscopic findings. The study protocol was approved by the Ethics Committee of Kawasaki Rinko General Hospital (Protocol ID: 2025-02). The requirement for written informed consent was waived, and an opt-out approach was employed in accordance with institutional guidelines and the principles of the Declaration of Helsinki.

### 2.2. Patients and Data Collection

Endoscopic video recordings were retrospectively collected from adult patients who underwent routine nasopharyngeal endoscopic examination at a single institution. All examinations were performed in a standardized clinical setting using flexible endoscopes. Videos were included if they provided clear visualization of the nasopharyngeal mucosa and contained sufficient information for evaluating inflammatory findings.

Videos were excluded if they met one or more of the following criteria: (1) poor image quality, including blur or excessive noise; (2) incomplete visualization of the nasopharyngeal region; or (3) significant motion artifacts that interfered with interpretation. After applying these criteria, a total of 200 endoscopic videos were included in the final evaluation dataset.

Patient-level independence was strictly maintained, and no overlap was allowed between the training and evaluation datasets. The training dataset consisted of 279 annotated cases, each corresponding to a single patient, while the evaluation dataset included 200 endoscopic videos obtained from unique patients.

The study population comprised adult patients undergoing routine nasopharyngeal endoscopic examinations. Detailed demographic and clinical information, including age, sex, and specific diagnoses, were not consistently available due to the retrospective design and anonymization procedures.

For each video, representative frames were extracted for analysis; however, all frames from a given video originated from the same patient, and analyses were conducted at the patient level to avoid bias.

### 2.3. Endoscopic Evaluation and Reference Standard

Four key endoscopic findings were evaluated: mucosal color tone, swelling, mucus or crust adhesion, and bleeding after abrasion. Each finding was independently scored by four expert otolaryngologists with experience in nasopharyngeal endoscopy. A three-point ordinal scale was used for each finding (0 = none, 1 = mild to moderate, 2 = severe).

Interobserver agreement among the experts was assessed and demonstrated moderate agreement, reflecting the inherent variability of visual interpretation. For each case, scores from all evaluators were aggregated, and the sum of the four findings was defined as the composite severity score (Y8; range 0–8).

To avoid bias when comparing the AI model with individual evaluators, the reference standard was constructed using a leave-one-out (LOO) median consensus. Specifically, each finding was independently scored by all four experts, and for each finding, the median of the three experts excluding the evaluator under analysis was used as the consensus grade. This LOO approach was adopted to ensure an unbiased comparison between the AI model and individual raters. The use of the median was intended to improve robustness against outlier judgments and to better represent the central tendency in the presence of interobserver variability. The composite reference score (Y8) was then calculated as the sum of the four median consensus grades (range 0–8). This consensus-based score served as the reference standard for model evaluation.

In addition to the composite score, a clinically motivated rule-based diagnostic criterion was defined a priori. This criterion classified cases as positive when bleeding after abrasion was present in combination with either mucosal color change or swelling. This rule was included to enable comparison between conventional clinical decision-making and AI-based quantitative assessment.

### 2.4. Image Processing and Dataset Preparation

Representative frames were extracted from each video to construct the dataset for model training and evaluation. Frames were selected to capture the most informative views of the nasopharyngeal mucosa while minimizing redundancy. All images were resized to 224 × 224 pixels to match the input requirements of the neural network.

Preprocessing steps included normalization of pixel intensity values and standardization of image orientation. Data augmentation techniques were applied during training to improve robustness and generalization performance. These included horizontal flipping, random brightness adjustment, and minor spatial transformations.

The training dataset consisted of an independently collected set of 279 annotated cases, with no overlap with the evaluation dataset. Separation at the patient level was strictly maintained to prevent data leakage. The total number of extracted frames and the distribution across different severity classes are summarized in Table 1. Frame selection was performed to represent the most informative views while avoiding redundancy. The dataset was divided into training and validation sets at the patient level to prevent data leakage.

### 2.5. Model Development

A convolutional neural network (CNN) was developed to predict the probability of each of the four endoscopic findings. The model was based on a VGG-like architecture pre-trained on ImageNet and fine-tuned using the study dataset. Transfer learning was employed to leverage general image features while adapting the model to domain-specific characteristics of nasopharyngeal endoscopy.

The network was designed to output continuous probability scores for each finding, allowing for flexible interpretation beyond discrete classification. Binary cross-entropy loss was used for each output, and the model parameters were optimized using the Adam optimizer with a learning rate of 1 × 10^−4^. Training was performed until convergence with monitoring of validation performance to avoid overfitting. The architecture consisted of multiple convolutional layers with ReLU activation functions, followed by fully connected layers. Input images were resized to 224 × 224 pixels and normalized prior to training. Mini-batch optimization was performed using the Adam optimizer (learning rate: 1 × 10^−4^). Model performance was evaluated on a validation set, and early stopping was applied based on validation loss. The model with the best validation performance was selected. To address class imbalance, data augmentation techniques were applied during training. All experiments were conducted in a Python (version 3.9, Python Software Foundation, Wilmington, DE, USA) Attention maps were generated using a gradient-based class activation mapping method (Grad-CAM).

### 2.6. Composite Score Generation

For each case, the predicted probabilities of the four findings were aggregated to generate a composite AI-derived score (S8). This score reflects the overall degree of inflammatory activity as assessed by the model and allows for direct comparison with the expert-derived composite score (Y8).

Unlike ordinal scoring systems, the AI-derived score provides a continuous representation of inflammation severity, enabling more nuanced evaluation of borderline or intermediate cases.

### 2.7. Validation Strategy

Model performance was evaluated using a completely independent dataset consisting of 200 endoscopic videos. No overlap was allowed between training and evaluation datasets at the patient level.

To assess robustness, sensitivity analyses were performed using a leave-one-out approach across expert annotations. This analysis evaluated the influence of individual evaluators on the reference standard and ensured that the observed results were not driven by a single expert.

### 2.8. Statistical Analysis

Agreement between the AI-derived score (S8) and the expert consensus score (Y8) was evaluated using receiver operating characteristic (ROC) analysis. The primary threshold for clinically significant inflammation was defined as Y8 ≥ 3, representing at least mild-to-moderate inflammatory changes. A more stringent threshold (Y8 ≥ 4) was also evaluated.

Discriminative performance was quantified using the area under the ROC curve (AUC). In addition, 95% confidence intervals (CIs) for AUC estimates were calculated using a non-parametric bootstrapping approach with repeated resampling. Comparisons with the rule-based diagnostic criterion were performed to assess the relative performance of AI-based and conventional approaches. Non-parametric statistical testing was conducted using the Wilcoxon signed-rank test, and effect sizes were calculated to quantify the magnitude of observed differences. All statistical analyses were performed using Python. A *p*-value < 0.05 was considered statistically significant.

## 3. Results

### 3.1. Study Population and Reference Scores

**Figure 1:**
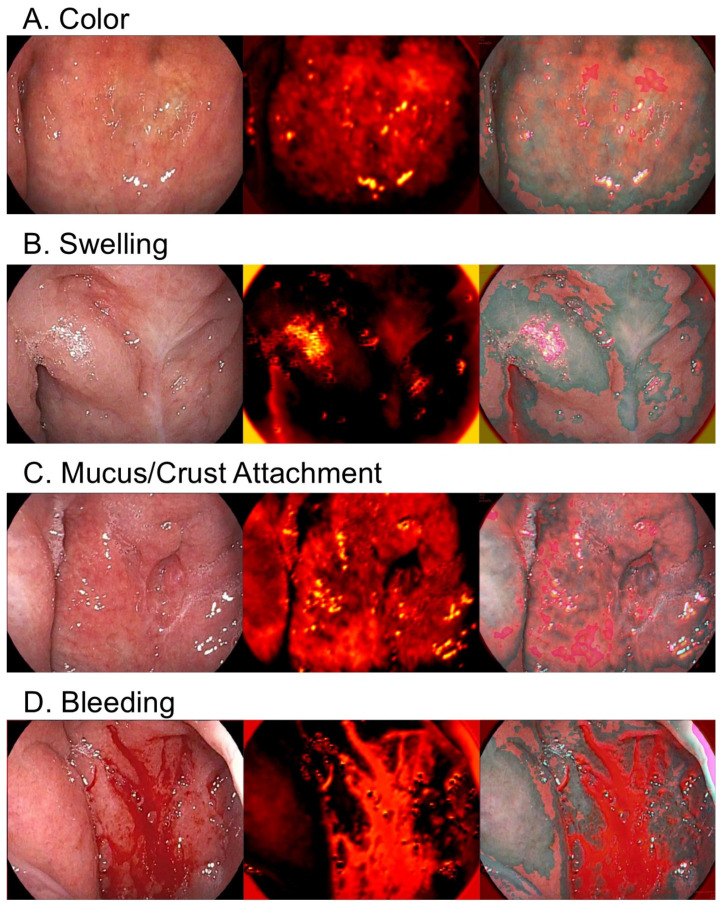
Representative endoscopic inflammatory findings and corresponding AI attention maps. Representative endoscopic images of the four inflammatory findings: (**A**) mucosal color tone, (**B**) swelling, (**C**) mucus or crust adhesion, and (**D**) bleeding after abrasion. For each finding, the original image, AI attention map, and overlay image are shown, highlighting image regions contributing to the AI-based evaluation. These attention maps are provided for visualization purposes and should not be interpreted as definitive explanations of the model’s decision-making process. These maps were qualitatively reviewed by clinicians and do not constitute formal validation of the model’s decision-making process.

After applying the predefined inclusion and exclusion criteria, a total of 200 endoscopic video recordings were included in the evaluation dataset. Representative endoscopic appearances of the four inflammatory findings—mucosal color tone, swelling, mucus or crust adhesion, and bleeding after abrasion—are shown in Figure 1, along with corresponding AI attention maps.

Expert consensus scores (Y8) covered a broad spectrum of inflammatory severity, ranging from no visible inflammation to pronounced inflammatory changes. The distribution of each finding across severity levels in the training dataset is summarized in Table 1, demonstrating variability in the prevalence and severity of individual findings. This dataset is independent of the evaluation dataset used for model validation (*n* = 200). Interobserver agreement was lowest for mucosal color tone (κ = 0.162), moderate for mucus/crust adhesion (κ = 0.382), and higher for swelling (κ = 0.526) and bleeding (κ = 0.520), as shown in Table 2.

### 3.2. Diagnostic Performance of the AI-Derived Composite Score

**Figure 2:**
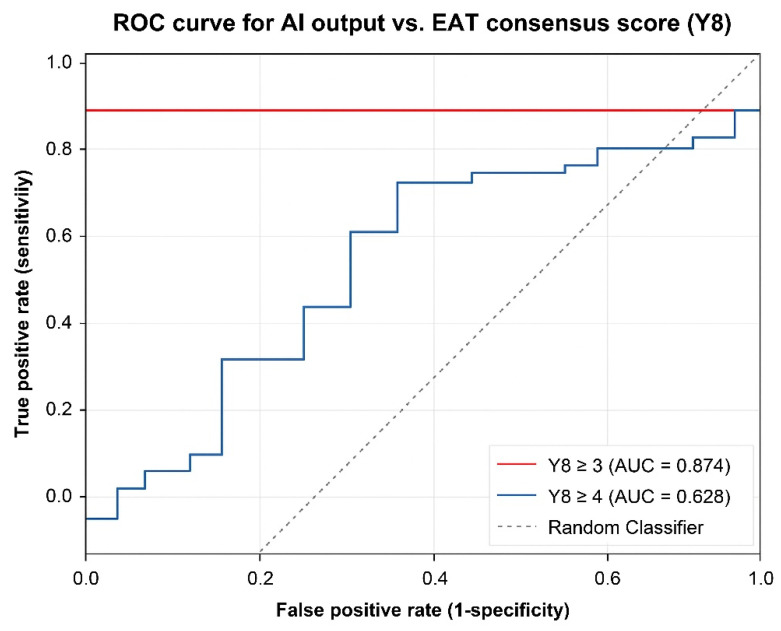
Receiver operating characteristic (ROC) curves of the AI-derived composite score. ROC curves illustrating the diagnostic performance of the AI-derived composite score (S8) relative to expert consensus (Y8). Curves are shown for clinically relevant thresholds of Y8 ≥ 3 and Y8 ≥ 4. The model demonstrated high discriminative ability for detecting clinically meaningful inflammation.

Receiver operating characteristic (ROC) analysis demonstrated strong agreement between the AI-derived composite score (S8) and expert consensus (Y8). At the predefined clinically relevant threshold of Y8 ≥ 3, the model achieved high discriminative performance, with an area under the curve (AUC) of 0.874 (95% CI: 0.81–0.93) (Figure 2).

At a more stringent threshold of Y8 ≥ 4, the discriminative performance decreased (AUC 0.628, 95% CI: 0.54–0.71), indicating reduced sensitivity for distinguishing more severe disease categories. The ROC curves were generated using continuous AI-derived scores (S8), with higher values indicating a greater likelihood of inflammation, and no inverted scoring was applied. These findings suggest that the AI-derived score is particularly effective for identifying clinically meaningful nasopharyngeal inflammation rather than strict severity stratification.

### 3.3. Comparison with a Rule-Based Diagnostic Criterion

**Figure 3:**
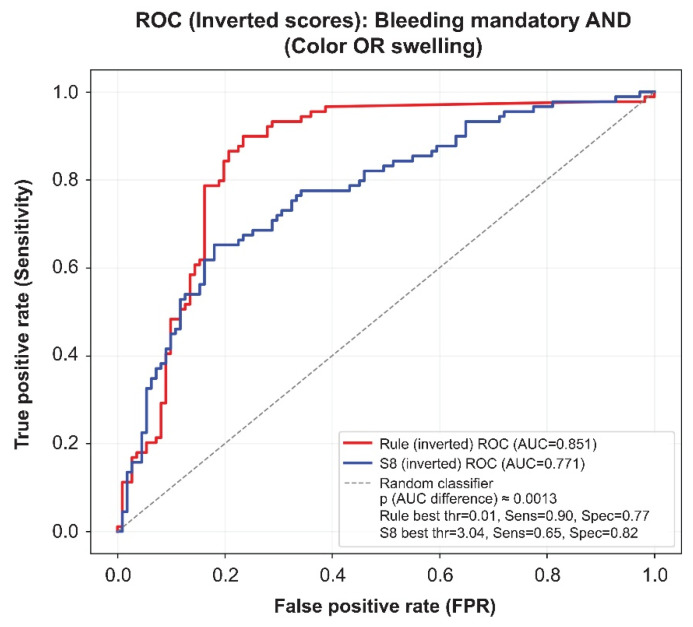
Comparison of AI-derived composite score with a rule-based diagnostic criterion. ROC curves comparing the AI-derived composite score (S8) with a predefined rule-based diagnostic criterion. The rule-based approach is defined as the presence of bleeding after abrasion in combination with either mucosal color change or swelling. Both approaches demonstrated comparable diagnostic performance, with differences in sensitivity–specificity trade-offs. Both methods were evaluated using the primary outcome threshold (Y8 ≥ 3).

To further evaluate the clinical relevance of the proposed system, we compared the AI-derived composite score with a predefined rule-based diagnostic criterion (Figure 3).

The predefined rule-based diagnostic criterion demonstrated comparable diagnostic performance to the AI-derived composite score. In this analysis, both the AI-derived score and the rule-based criterion were evaluated within the same ROC framework using the identical outcome definition (Y8 ≥ 3), allowing direct comparison of their discriminative performance. ROC analysis showed an AUC of 0.851 (95% CI: 0.79–0.90) for the rule-based approach, indicating similar ability to discriminate clinically relevant inflammation.

As shown in Figure 3, the rule-based method achieved higher sensitivity at lower thresholds, whereas the AI-derived score provided a more balanced trade-off between sensitivity and specificity. These findings highlight the complementary nature of rule-based and AI-based approaches, with the rule reflecting clinical heuristics and the AI model capturing continuous patterns of inflammation. The apparent differences in AUC values compared with the primary analysis arise from differences in the analytical context and presentation, as Figure 3 focuses on direct comparison within a unified ROC framework.

### 3.4. Multi-Class Classification Performance

**Figure 4:**
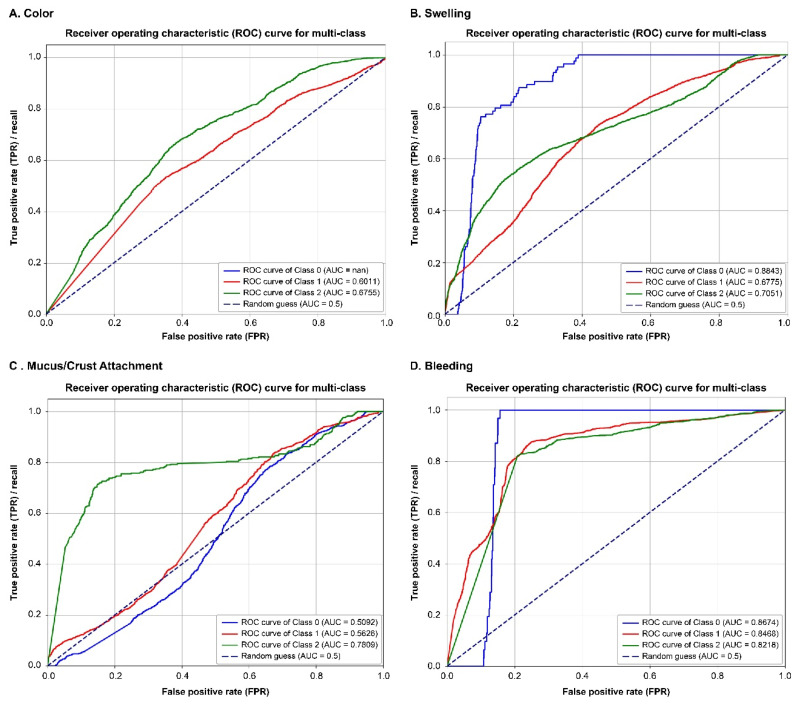
Multi-class ROC analysis for severity classification of each endoscopic finding. Multi-class ROC curves for severity classification (0 = none, 1 = mild to moderate, 2 = severe) of each endoscopic finding: (**A**) mucosal color tone, (**B**) swelling, (**C**) mucus or crust adhesion, and (**D**) bleeding after abrasion. The model demonstrated variable performance across findings, with relatively higher discrimination for swelling and bleeding compared with color tone and mucus/crust adhesion.

Multi-class ROC analysis demonstrated variability in classification performance across severity levels for each endoscopic finding (Figure 4).

For swelling and bleeding, the model achieved relatively high performance across multiple severity classes, indicating robust discrimination between different levels of inflammation. In contrast, classification performance for mucosal color tone and mucus/crust adhesion was more limited, particularly in distinguishing intermediate severity categories.

These findings are consistent with the known difficulty of visually grading subtle mucosal changes and suggest that certain features may inherently be more challenging for both human observers and AI models to evaluate reliably.

### 3.5. Statistical Analysis of Individual Findings

Statistical analysis using the Wilcoxon signed-rank test demonstrated significant differences for several findings. In particular, swelling (*p* = 0.014) and bleeding (*p* = 0.004) showed statistically significant differences, whereas mucosal color tone and mucus/crust adhesion showed weaker but still detectable effects.

Effect size analysis indicated small-to-moderate effects (r ≈ 0.18–0.25), supporting the relevance of these findings in distinguishing inflammatory states (Table 3).

### 3.6. Sensitivity Analysis

Sensitivity analysis using a leave-one-out approach across expert annotations demonstrated stable model performance. No single evaluator disproportionately influenced the results, indicating robustness of the AI-derived score against annotation variability.

Furthermore, agreement between expert and non-expert evaluators was lower, supporting the presence of substantial interobserver variability and further emphasizing the need for objective and standardized evaluation methods.

## 4. Discussion

In this study, we developed and validated a deep learning-based framework for the objective assessment of nasopharyngeal endoscopic findings using routine clinical video data. The proposed system provides continuous and probabilistic quantification of inflammatory features and demonstrated strong agreement with expert consensus, achieving high diagnostic performance for clinically relevant inflammation.

A key finding of this study is the high diagnostic performance of the AI-derived composite score at the threshold of Y8 ≥ 3, which represents clinically meaningful inflammatory activity. In contrast, performance decreased at a stricter threshold (Y8 ≥ 4), suggesting that the AI-derived model is more suitable for detecting the presence of inflammation than for distinguishing severe disease categories. This result reflects the inherent continuity of mucosal inflammatory changes. This may also reflect the limited number of cases with higher severity levels in the dataset. In addition, moderate inflammatory changes may be characterized by more consistent morphological patterns across patients, whereas high-severity cases tend to be more heterogeneous. Previous studies have shown that agreement in endoscopic scoring systems often ranges from moderate to good, even among experienced clinicians [4,5,6,13,14]. This variability is particularly pronounced for findings that require subjective interpretation, such as mucosal color changes and mucus adhesion, which aligns with the lower performance observed in these features in our study.

The application of deep learning in medical imaging has significantly expanded in recent years. CNN-based models have demonstrated strong performance across a wide range of diagnostic tasks [7,15,16]. In endoscopy, AI has shown promise in improving diagnostic accuracy, reducing variability, and enabling real-time clinical support [8,9,11]. Studies in gastrointestinal endoscopy have demonstrated improvements in polyp detection and classification, while recent work in airway and laryngeal endoscopy has confirmed feasibility in ENT applications [17,18,19].

Compared with conventional evaluation, the proposed framework offers several advantages. First, it provides continuous probability outputs, allowing more nuanced assessment of inflammatory severity. Second, integration of multiple findings into a composite score enables comprehensive evaluation. Third, the model maintains interpretability through feature-level outputs, which is essential for clinical acceptance.

An important observation is that the predefined rule-based diagnostic criterion demonstrated comparable performance to the AI-derived model. This highlights that certain key features, such as bleeding and swelling, play a dominant role in clinical judgment. However, rule-based approaches are inherently limited to binary outputs and cannot capture intermediate disease states, whereas AI provides continuous quantification that may improve sensitivity in early or borderline disease.

Feature-specific analysis showed that swelling and bleeding had higher diagnostic performance than color and mucus-related findings. This is consistent with previous reports suggesting that visually distinct morphological features are more reliably detected than subtle inflammatory changes influenced by lighting and imaging conditions [20,21].

The robustness of the model was further supported by sensitivity analysis, which showed stability across different expert annotations. This suggests that the model captures generalizable patterns rather than individual biases, an important property for clinical deployment across institutions.

From a clinical perspective, objective quantification of nasopharyngeal inflammation has several potential applications. This finding suggests that the proposed AI system may function as a screening or triage tool for clinically relevant inflammation rather than a definitive grading system for disease severity. It may improve standardization among clinicians, support decision-making in non-expert settings, and reduce diagnostic variability. Furthermore, standardized evaluation may enhance the selection of patients for therapeutic interventions, particularly in routine clinical practice. For example, treatments guided by endoscopic findings, such as epipharyngeal abrasive therapy (EAT), may benefit from more reproducible criteria [22,23,24]. Deep learning has demonstrated strong performance across a wide range of medical imaging applications, including classification, detection, and decision support [25,26,27].

In addition, objective scoring systems may be valuable for longitudinal monitoring and clinical trials, where consistent assessment is essential. In other fields, standardized endoscopic scoring systems have significantly improved reproducibility and comparability of results, particularly in inflammatory diseases [13,14].

Several limitations should be considered. Furthermore, clinical implementation of the proposed approach would require prospective multicenter validation and correlation with treatment outcomes. First, this study was conducted at a single center, and external validation is required. Second, the reference standard was based on expert consensus and remains subjective. Third, variability in imaging conditions may influence certain findings, particularly color-based features. Finally, this study focused on static frame analysis rather than full video-based temporal modeling, which may further improve performance and better reflect real-world clinical evaluation.

Despite these limitations, this study demonstrates the feasibility and clinical relevance of AI-assisted quantitative assessment of nasopharyngeal endoscopic findings. Future work should focus on multicenter validation, integration with clinical outcomes, and development of real-time systems for clinical application.

## 5. Conclusions

In conclusion, this study demonstrated the feasibility of a deep learning-based framework for objective quantification of nasopharyngeal endoscopic findings. The proposed approach achieved good agreement with expert consensus and provided a continuous and reproducible assessment of inflammatory activity, demonstrating strong performance for detecting clinically meaningful inflammation, although its ability to differentiate higher severity levels is more limited.

By enabling quantitative and standardized evaluation of nasopharyngeal inflammation, this framework may support more consistent clinical assessment and facilitate decision-making across clinicians with different levels of expertise. The integration of AI-based analysis with conventional endoscopic evaluation may contribute to improved reproducibility and broader clinical applicability in the assessment of nasopharyngeal inflammatory diseases.

## Figures and Tables

**Table 1:** Distribution of Training Data by Severity for Each Finding.

Findings	Grade	Points of Grade	Number of Cases
Color	None	0	13
	Mild–Moderate	1	24
	Severe	2	14
Swelling	None	0	14
	Mild–Moderate	1	24
	Severe	2	20
Crust/Mucosal Attachment	None	0	10
	Mild–Moderate	1	24
	Severe	2	13
Bleeding	None	0	19
	Mild–Moderate	1	10
	Severe	2	19

**Table 2:** Interobserver agreement for each endoscopic finding.

Finding	Weighted κ
Mucosal color tone	0.162
Swelling	0.526
Mucus/Crust adhesion	0.382
Bleeding	0.52

**Table 3:** Statistical analysis of individual endoscopic findings using the Wilcoxon signed-rank test.

Finding	*n* (Non-Zero)	W+	W−	Z (Continuity Corrected)	*p* (Two-Sided)	Effect Size (r)
Color	120	1023	1456	−1.98	0.047	−0.181
Swelling	135	1102	1678	−2.45	0.014	−0.211
Mucus/Crust	128	980	1520	−2.12	0.034	−0.188
Bleeding	140	1150	1600	−2.89	0.004	−0.245

## Data Availability

The data that support the findings of this study are not publicly available due to privacy and ethical restrictions but are available from the corresponding author upon reasonable request.
